# The histone deacetylase inhibiting drug Entinostat induces lipid accumulation in differentiated HepaRG cells

**DOI:** 10.1038/srep28025

**Published:** 2016-06-20

**Authors:** Abigail D. G. Nunn, Tullio Scopigno, Natalia Pediconi, Massimo Levrero, Henning Hagman, Juris Kiskis, Annika Enejder

**Affiliations:** 1 Center for Life Nano Science@Sapienza, Istituto Italiano di Tecnologia, Viale Regina Elena 291, 00161 Rome, Italy; 2 Department of Physics, Sapienza University of Rome, Piazzale Aldo Moro 5, 00185 Rome, Italy; 3 Department of Molecular Medicine, Sapienza University of Rome, Viale Regina Elena 291-293, 00161 Rome, Italy; 4 Department of Internal Medicine, Sapienza University of Rome, Piazzale Aldo Moro 5, 00185 Rome, Italy; 5 INSERM U1052- Cancer Research Center of Lyon (CRCL), 151 Cours Albert Thomas, 69008 Lyon, France; 6 Department of Hepatology, Croix Rousse Hospital, Hospices Civils de Lyon, Lyon 69004, France; 7 Department of Biology and Biological Engineering, Chalmers University of Technology, SE-412 96 Gothenburg, Sweden

## Abstract

Dietary overload of toxic, free metabolic intermediates leads to disrupted insulin signalling and fatty liver disease. However, it was recently reported that this pathway might not be universal: depletion of histone deacetylase (HDAC) enhances insulin sensitivity alongside hepatic lipid accumulation in mice, but the mechanistic role of microscopic lipid structure in this effect remains unclear. Here we study the effect of Entinostat, a synthetic HDAC inhibitor undergoing clinical trials, on hepatic lipid metabolism in the paradigmatic HepaRG liver cell line. Specifically, we statistically quantify lipid droplet morphology at single cell level utilizing label-free microscopy, coherent anti-Stokes Raman scattering, supported by gene expression. We observe Entinostat efficiently rerouting carbohydrates and free-fatty acids into lipid droplets, upregulating lipid coat protein gene *Plin4*, and relocating droplets nearer to the nucleus. Our results demonstrate the power of Entinostat to promote lipid synthesis and storage, allowing reduced systemic sugar levels and sequestration of toxic metabolites within protected protein-coated droplets, suggesting a potential therapeutic strategy for diseases such as diabetes and metabolic syndrome.

Acetylation of lysine residues in proteins has emerged as a ubiquitous mechanism for reversible, post-translational modification of protein functionality and activity, being modulated by two counteracting types of enzymes in response to cellular signalling: histone acetyltransferases (HATs) and histone deacetylases (HDACs). These were first discovered as key actors in the epigenetic machinery, modulating gene expression by altering the acetylation of histones in chromatin affecting the compactness of the DNA-histone complex and hence the binding of transcription factors^1^,^2^. However, it soon became clear that the functionality of many non-histone proteins located in the nucleus was also modulated by their acetylation pattern, such as proteins involved in pathways controlling apoptosis, cell cycle progression, redox pathways, mitotic division, DNA repair, and cell migration. Not so surprisingly, altered expression of HATs and HDACs has been reported in association with a number of human diseases such as cancer, inflammatory and neurodegenerative diseases^3^.

Recently, HDACs have also been shown to modulate metabolic pathways in the liver; inhibition upregulates genes coding for *de novo* lipogenesis and lipid sequestration together with down-regulating gluconeogenesis^4^,^5^. Improved tolerance to glucose and higher insulin sensitivity were reported, suggesting HDAC inhibition as a promising target for therapeutic intervention in diabetes, metabolic syndrome and related diseases. Hepatic lipid accumulation is, in long-term perspective, harmful for the functionality of the liver, but at over-nutrition it has been shown to have a beneficial impact by safely sequestering excess toxic metabolites in perilipin-coated lipid droplets, clearing cellular free-fatty acids and intermediates that would otherwise cause damage to insulin signalling and create cardiovascular risk^6^. These new aspects of lysine acetylation as a metabolic modulator motivate studies of the impact of synthetic HDAC inhibitors on hepatic lipid metabolism: such drugs can potentially offer novel therapeutic solutions for diseases that affect a large proportion of the population and they reside among the most promising classes of drugs currently under clinical evaluation.

In this work we study the effect of the HDAC inhibitor Entinostat (MS275), which is already in clinical trials for cancer treatment, on hepatic lipid metabolism in HepaRG cells by quantifying lipid store droplets and their distribution at single cell level utilizing a label-free microscopy technique, coherent anti-Stokes Raman scattering (CARS). HepaRG is a human liver cell line that upon differentiation exhibits characteristics of primary human hepatocytes and therefore offers a good representation of human hepatic xenobiotic metabolism^7^. CARS microscopy, like Raman microscopy^8^, enables chemically specific label-free image contrast for cellular lipids. Use of the strong CH_2_ symmetric stretching vibration at around 2840 cm^−1^, in a coherent Raman excitation scheme allows pico(10^−12^)-gram sensitivity and sub-micron spatial resolution at high speeds, avoiding problems that can be associated with the use of perturbatively large fluorescent labels or stains^9^,^10^. Here we present analysis, using three-dimensional and subcellular spatial information from CARS images, of the effect of the HDAC inhibiting drug Entinostat, comparing differentiated HepaRG (dHepaRG) cells that have undergone three treatments: i, Entinostat, ii, fatty acid overload (sodium oleate) and iii, Entinostat and fatty acid overload in combination, as well as a control. Addition of oleate salts to the cell culture medium allows rapid access to lipids without need of *de novo* fatty acid synthesis, inducing lipid storage by enzymatic regulation, which we compared with the lipid accumulation mechanisms observed after addition of the drug Entinostat, inducing *de novo* lipid synthesis via gene regulation. We reveal contrasting modes of lipid droplet loading for the treatments, correlating image statistics with gene expression data. We demonstrate that Entinostat induces the sequestration of lipid droplets with increased lipid coat proteins, highlighting the potentialities of Entinostat and inhibitors of HDACs in the treatment of metabolic syndrome.

## Results

**Figure 1:**
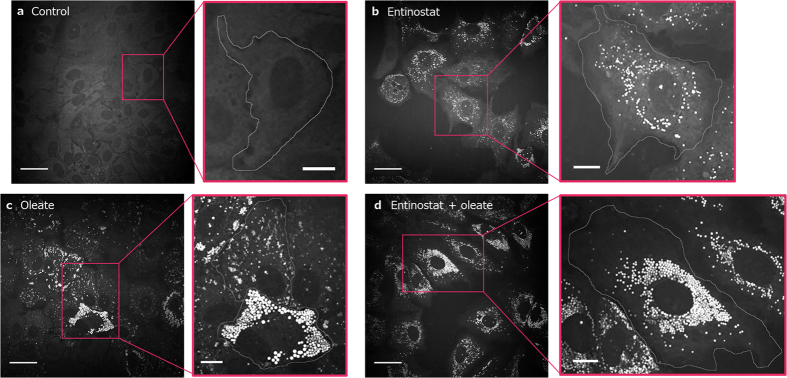
Different modes of lipid accumulation. Examples of CARS images of control cells (no treatment) (**a**), cells treated with Entinostat (**b**), cells treated with oleic acid overload (**c**), cells treated with both oleic acid overload and Entinostat (**d**). Entire fields of view are shown on the left, and insets with cell outlines overlaid on the right. Entinostat induces a moderate degree of lipid droplet accumulation across almost all cells (**b**), whereas with oleate (free fatty acid), there is significant increase in lipid accumulation in some cells, observed as higher numbers of lipid droplets per cell as well as larger volumes of individual droplets; two examples of different lipid structure accumulation are shown in the expansion in (**c**). Entinostat treatment under conditions of excess metabolite availability results in a strong lipid loading in almost all cells (**d**). Images are single planes taken from *z*-stacks, displayed after noise removal using PureDenoise (see Materials and Methods). Scale bars for images are 30 μm and 10 μm for expansions.

CARS images of cells exposed to different conditions (control, Entinostat treatment, oleate overload and Entinostat combined with oleate overload) show significantly different patterns of lipid accumulation (Fig. 1), in the absence of significant cell toxicity (Supplementary Fig. 1). All of the treatments (Fig. 1b–d) result in an increase in average lipid droplet numbers compared to the untreated cells (Fig. 1a). A quite uniform increase in lipid droplet numbers across all cells is apparent as a result of Entinostat intake (Fig. 1b), whereas for the oleate treated cells, the cell-to-cell variability is large: there appears a strong increase in the lipid levels in some cells, and a lesser increase in others (Fig. 1c). For the combined treatment very strong loading with lipid droplets is induced consistently in almost all cells (Fig. 1d).

### Lipid droplet numbers and volumes increase dramatically with treatment

**Figure 2:**
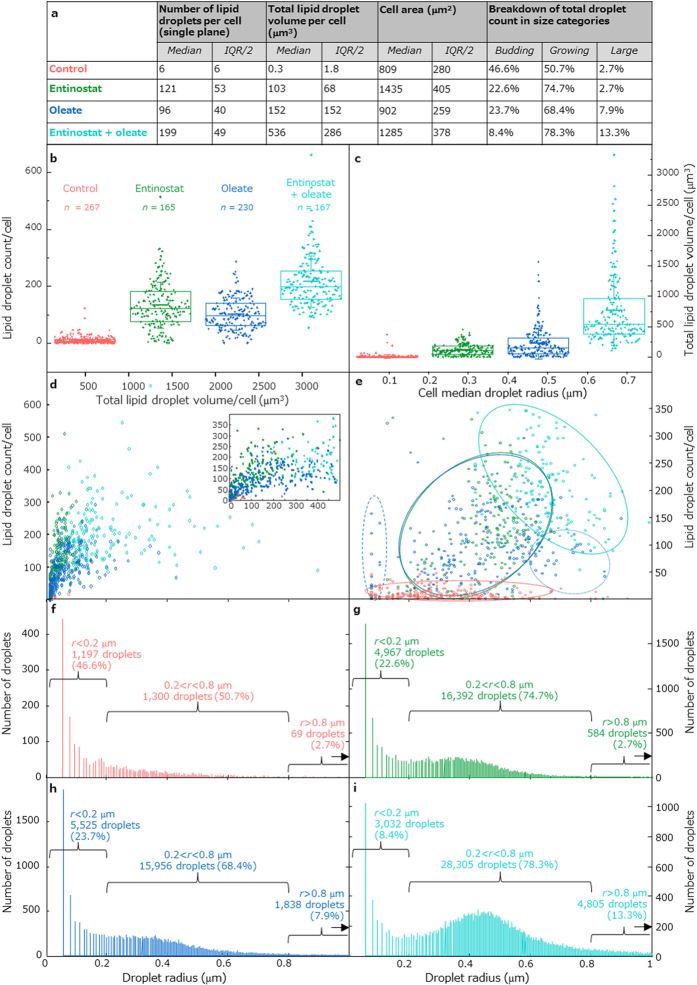
Results of quantitative image analysis of lipid droplet properties of HepaRG cells. Summary table showing the median and interquartile range for three cellular quantities derived from image analysis, and breakdown of droplet populations across all cells into size categories, budding (*r* < 0.2 μm), growing (0.2 < *r* < 0.8 μm) and large (*r* > 0.8 μm) (**a**). Box and whisker plots of the number of droplets per cell (**b**) and the total lipid volume per cell (**c**). Scatter of the total lipid droplet volume versus the lipid droplet count (**d**) shows that oleic acid enables the storage of large lipid volumes in fewer droplets, with or without co-incubation with Entinostat, also seen in the plot expansion inset. Median droplet radius versus the lipid droplet count (truncated, **e**) reveals three main groups, highlighted by the overlaid solid-lined circles: 1-untreated, 2-single treatments (either drug or oleate) and 3-the combined treatment; the higher degree of cell-to-cell variability for the non-drug treated cells is apparent in this plot, where two subgroups of the oleate single treatment are also circled, one with low droplet count and small median droplet radius (dashed line) and one with low droplet count and large median radius (dotted line); an approximate trend of increasing number of droplets with radius size for the single Entinostat treatment but the reverse relationship for the combined treatment could be related to droplet fusion levels. Truncated histograms showing the distribution of droplet radii from all cells, with the numbers of budding, growing and large droplets overlaid (**f–i**). For drug treatment an increase in lipid volume, as for the oleate treatment, occurs but along with a higher average number of droplets than the oleate treatment; for the combined treatment, the strong loading of the cells with lipids results in the highest number of droplets and much increased total lipid volume, manifesting as a substantial increase in the relative proportion of growing and large droplets. The colour scheme and cell counts for all plots is given in (**b**). For (**b**,**c**), thick horizontal bars represent means, boxes mark 25th, 50th and 75th percentiles, and whiskers indicate standard deviations.

Quantitative image analyses of the lipid droplet volumes and areas based on a statistically significant number of cells per treatment category are summarized in Fig. 2a. In Fig. 2b,c we see that the number of droplets per cell, as well as the total lipid volume per cell, increases strongly with either treatment by Entinostat or oleate overload compared to the control cells, and further increases with the combined treatment of drug and oleate. Total lipid volumes for the different treatments acquired from the CARS microscopy images are in agreement with measured lipid amounts from fluorescence activated cell sorting (FACS) analysis using bodipy (Supplementary Fig. 2). For both the number of droplets per cell and the sum of lipid droplet volumes per cell, Wilcoxon rank-sum tests between all the different cell treatment conditions result in rejection of the null hypothesis for every pair of treatments, including the drug only and oleate only pair, indicating that the treatment populations vary significantly in these two statistical properties, at the 1% significance level. The median number of droplets per cell for the combined treatment is 199, and for the Entinostat and oleate single treatments it is 121 and 96 respectively, implying an approximately additive effect for the treatments. However, the effect for total lipid volume per cell is not additive: the median volume for the combined treatment is 536 μm^3^, for Entinostat only it is 103 μm^3^, and for oleate it is 152 μm^3^.

### Lipid droplet size variation

Statistically, a greater cell-to-cell uniformity of lipid droplet accumulation with Entinostat compared to oleate overload is highlighted in the cluster plot in Fig. 2e, where we show the populations as cellular median droplet radius versus droplet count per cell: the larger variation of median droplet radius across the cell population for the oleate treatment is apparent. We highlight three main groups for this cell population: one group (the largest) that occupies the same space in the scatter plot as the Entinostat treated cells, with intermediate median cell radii (highlighted in the plot by a solid-lined blue circle), another with small median droplet radii (dashed blue circle), and another with high values of median droplet radii (dotted blue circle). The population that was treated by the drug and oleate overload maintains the uniformity of the drug treatment but is shifted to significantly larger median droplet size and higher droplet count. The main populations of the Entinostat and oleate individual treatments show a trend of increasing number of lipid droplets per cell with increasing median droplet radius, indicating a population of growing lipid droplets in the cells. In contrast, the combined treatment shows an approximate trend of reduced number of lipid droplets with increasing median droplet radius, which could perhaps indicate that this population is in a state in which lipid droplet fusion is dominant. In Fig. 2f–i the bimodal distributions of radii of all lipid droplets for the different treatments are shown. They have a symmetric, almost Gaussian-like, peak shape between 0.2 and 0.8 μm, accompanied by a peak representing droplets with a radius <0.2 μm. The peaks at low values indicate the presence of budding lipid droplets, which are probably formed as precursors to other droplets^11^,^12^. For the control cells, the median (and half interquartile range) value of the droplet radii is 0.22(0.15) μm. Although the effect of the drug and of the oleate overload (Fig. 2g,h) seems at first glance, in these truncated plots, to be similar in terms of the droplet size distribution, with a peak at values of radius ~0.4 μm in both cases, if we define three different categories of droplet sizes based on the structures of the distributions (budding, *r* < 0.2 μm; growing 0.2 < *r* < 0.8 μm; large, *r* < 0.8 μm), looking at the percentage of larger droplets (*r* > 0.8 μm, also beyond the truncated histogram plots) we notice that this population is smaller for Entinostat treatment. So, with carbohydrates as energy source (Entinostat treatment), the proportion of these large droplets remains at 2.7%, as for the control, compared to fatty acid treatment in which the percentage is approximately tripled to 7.9%. For the combined treatment (Fig. 2i), the huge increase in total lipid volume can be described by two effects: (i) an increasing population of growing droplets (0.2 < *r* < 0.8 μm, 78.3%), all of which slightly larger, as evidenced by the shift in the peak of the radii distribution to ~0.45 μm, and (ii) a substantial increase in the number of large droplets (*r* > 0.8 μm, 13.3%).

### Droplet fusion and hints about growth dynamics

In Fig. 2i, the larger proportion of large lipid droplets in the combined treatment population (*r* > 0.8 μm, 13%) might result from fusion of individual smaller droplets. Studies by Boström *et al*. indicate that lipid droplets with larger size can result from fusion of small droplets, typically arriving towards the centre of the nucleus from the outer regions of the cell and then merging with neighbours^13^. Figure 2e provides some clues to the dynamics of lipid droplet growth in our cell study; we see that the cells for the combined treatment occupy a region of approximately negative trend, the more lipid droplets, the smaller the median droplet size and *vice versa*, by contrast, the Entinostat only treatment has an approximately positive trend such that more lipid droplet counts in a cell means a larger median droplet size. This suggests that the two treatment populations might have been imaged at two different stages of growth: in the drug only, the population is still “growing” overall, at the end of the 4 day incubation period, so that most of the droplets continued to increase in size probably by *in situ* triglyceride synthesis, whereas in the combined treatment, the cells are likely to be already saturated with lipids, so perhaps to conserve space or phospholipid membrane molecules, significant numbers of droplet fusion events have taken place after the individual expansion stage^12^.

### Subcellular droplet location and *in situ* triglyceride synthesis

**Figure 3:**
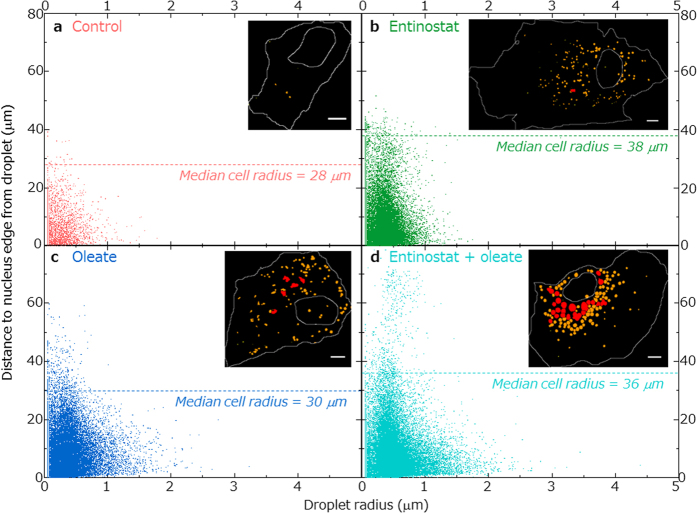
Intracellular droplet dispersion. Scatter plots of distance to nucleus edge from droplet centroid versus droplet radius for all droplets in entire cell sample reveal a slight trend of inverse relationship of droplet size with distance from nucleus in control (**a**), Entinostat (**b**) and oleate (**c**) cells; for Entinostat and oleate treatment (**d**), this trend becomes more distinct in some cells, as shown inset. For this combined treatment, increased numbers of medium-sized droplets results in more dispersion of these droplets away from the nucleus but the average droplet distance from the nucleus is greatest for the oleate treatment alone; the median cell radii dimension is shown as a dashed horizontal line on each subplot, which helps to highlight the greater dispersion within the cells – more droplets further from the nucleus – associated with fatty acid overload compared to treatment with only Entinostat. Insets qualitatively illustrate the different loadings of budding (*r* > 0.2 μm, yellow), growing (0.2 < *r* < 0.8 μm, orange) and large (*r* > 0.8 μm, red) droplets for example cells, and the difference in their locations relative to the overlaid cell boundary and nuclear edge, with more dispersion for oleate treatment apparent (scale bars 5 μm).

Statistical spatial analysis over the whole imaged cell population reveals that the median distance between lipid droplets and cell nucleus is larger for the oleate treated cells (8.0 μm) than it is for the other treatments (6.4 and 6.8 μm for the Entinostat treated and Entinostat and oleate treated cells respectively); this is despite the median nuclear radius being similar for all three samples (6.7, 6.4 and 6.5 μm for Entinostat, oleate, and combined treatments respectively), and the median cell area being considerably *smaller* for the oleate treated cells compared to the others (990 versus 1484 and 1427 μm^2^ for Entinostat and the combined treatments respectively, see Supplementary Figs 3 and 4 for more details of nuclear and cell sizes). Scatter plots showing the full distributions (truncated in Fig. 2f–i), of the lipid droplets’ sizes and their distances from the nucleus edge are shown in Fig. 3. These plots show the populations of lipid droplets from all cells, illustrating the substantial and similar collections of larger lipid droplets (*r* > 0.8 μm) for the categories with access to excess fatty acids. Figure 3 also reveals a slight negative trend of droplet size and distance from the nucleus – larger droplets (*r* > 0.8 μm) in all treatment cases and the control can be seen nearer the nucleus. This trend becomes more pronounced in the case of the combined treatment, where in some cells (such as the example illustrated in the inset of Fig. 3d) we see a negative gradient of droplet dimension as the distance from the nucleus increases. We can also see from this plot that the combined treatment of Entinostat and oleate overload induces the spreading of the population of medium-sized droplets to much larger (50–70 μm) distances from the nucleus. However, as noted above, the average location of the droplets in this treatment case is still nearer the nucleus than for the oleate overload treatment alone; the population at longer distances is only a small outgrowth of the main peri-nuclear droplet population. Relative dispersions of the droplets within the cell are illustrated by examples shown inset in Fig. 3.

Wilfling *et al*. recently reported the existence of two different classes of lipid droplets, “static” and “expanding” lipid droplets (sLDs and eLDs), that were observed after treatment of cells with oleic acid. eLDs were found to contain an important enzyme for triglyceride biosynthesis (GPAT4), which relocated to the droplets from the endoplasmic reticulum (ER) after the addition of oleic acid. So-called eLDs are therefore able to expand rapidly under the conditions of excess fatty acid by *in situ* triglyceride synthesis, whereas the sLDs remained a smaller uniform size, presumably because they had no access to the ER and therefore also to the triglyceride synthesis enzymes^14^. This pattern of behaviour is echoed in our images, where, as seen in Fig. 3, the overall trend is for larger lipid droplets always to be in peri-nuclear locations, where the density of ER is greatest. Furthermore, we can hypothesize that the two different populations of droplets that we see in the cells, under all treatments, budding (*r* < 0.2 μm), and growing (0.2 < *r* < 0.8 μm) could be associated with these two types of static and expanding lipid droplets (Fig. 2f–i). In this context, the effect of Entinostat in maintaining, on average, droplet populations nearer the nucleus (median distance to nucleus of droplet is 6.4, 8.0 and 6.8 μm for Entinostat, oleate and combined treatments respectively, also see Fig. 3) implies, as we observe, a super-efficient capacity for uptake of the fatty acids and *in situ* conversion into triglycerides in the combined treatment because most of the droplets are within easy reach of the ER. This mechanism could contribute, alongside the droplet fusion hypothesis discussed above, to the presence of the large number of large droplets in the combined treatment. Furthermore, Kassan *et al*. report that the growth of lipid droplets is dependent on free-fatty acid concentration but the number of droplets is independent of this^15^, which agrees with our median cellular lipid volume increase in the combined treatment of nearly 2000-fold, but only a 30-fold increase in droplet numbers with respect to the control. A time-lapse imaging study of the effect of Entinostat on lipid droplet growth could elucidate further details of the dynamics of formation and growth, by fusion and/or by *in situ* triglyceride synthesis, and would also aid our understanding of the biomedical implications use of this drug, observing the effect of drug removal in cells as a model for understanding the longer-term effects in the liver of Entinostat after treatment is completed.

### Lipid droplet coat protein and *de novo* lipogenesis gene expression upregulated

**Figure 4:**
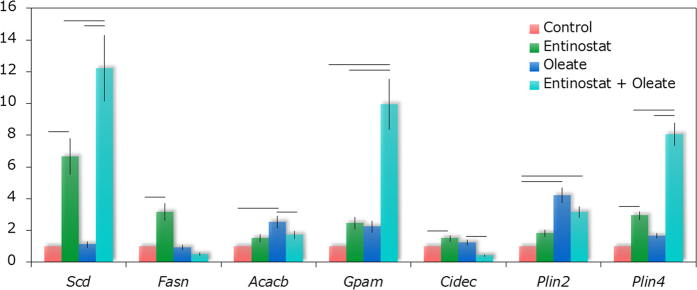
Results of Real-time PCR analysis. *De novo* lipogenesis (*Scd*, *Fasn*), triglyceride synthesis (*Acacb*, *Gpam*), and lipid sequestration (*Cidec*, *Plin2*, *Plin4*); data are expressed as fold induction (treated versus untreated). Free fatty acid (FFA, oleate) overload shows a minor impact at gene expression level, except for a ~four-fold upregulation of the gene that encodes for the protein Perilipin 2 (*Plin2*), which coats mature lipid droplets. Entinostat induces a significant upregulation of genes involved in *de novo* lipogenesis (*Scd*, *Fasn*) and the gene (*Plin4*) for the protein Perilipin 4, coating emerging lipid droplets. Finally, for Entinostat treatment with access to FFAs a combination of the upregulated genes (*Scd*, *Plin2* and *Plin4*) can be observed complemented by *Gpam*, inhibiting the transport of acyl-CoA into mitochondria hence promoting triglyceride synthesis. Each bar represents the mean of three independent experiments [horizontal bar marks *p*-value < 0.005].

To further understand the changes in patterns of lipid droplet formation in the context of the histone de-acetylase inhibition caused by Entinostat, we carried out a gene expression analysis on treated HepaRG cells (Fig. 4). It confirms the observations made by CARS microscopy on the lipid droplet morphologies and distributions; a significant upregulation of *Scd* and *Fasn* (*de novo* lipogenesis) can be noted combined with an upregulation of *Plin4*, related to the budding of lipid droplets^16^. Entinostat treatment at oleate overload approximately doubles the upregulation of *Scd* and *Plin4*, but also induces a manifest upregulation of *Gpam* (promoting triglyceride synthesis) as well as increased expression of *Plin2* (significant for mature lipid droplets). Oleate overload alone targets primarily the metabolic enzymes and shows a relatively small impact at gene expression level beside a four-fold upregulation of *Plin2* related to the formation of mature lipid droplets^16^,^17^,^18^.

The upregulation of both *Scd* and *Fasn*, genes for *de novo* FA synthesis, with Entinostat treatment are in full agreement with the work of Sun *et al*. in which HDAC3 depletion was clearly linked to a rerouting of metabolites towards lipid synthesis and away from glucose synthesis, with a resultant increase in insulin sensitivity^5^. *Scd* encodes the protein Stearoyl-CoA desaturase which is responsible for producing unsaturated oleic acid from saturated stearic acid, and *Fasn* encodes fatty acid synthase which produces the saturated fatty acid palmitate. We see upregulation of *Fasn* in the presence of Entinostat, only without free fatty acid (FFA) overload, hence the combined effect of the two treatments seems to be suppression of the synthesis of the saturated FA in favour of the unsaturated one. Overloading hepatic cells with oleate has been reported to induce down-regulation of lipogenesis genes including *Scd*, as per our results for the oleate incubation alone^19^. *Acacb* and *Gpam* are examples of genes associated with triglyceride synthesis and the inhibition of Acyl-CoA transference to mitochondria, and thus our observation of upregulation of these genes alongside lipid accumulation with all treatments (Fig. 4) indicates that fatty acid oxidation is dominated by fatty acid synthesis^5^.

Although expression of the gene in healthy liver cells is absent, *Cidec* (Cell Death-Inducing DFFA-Like Effector C), which codes for the lipid coat protein Fat-specific protein 27, has been observed in the liver after fasting or high-fat diet in mice, and its upregulation has been associated with large lipid droplets in adipocytes^20^,^21^. Here (Fig. 4) we see only a slight upregulation of *Cidec* by single treatments with oleate and or Entinostat, possibly as the amount of FFAs was not sufficient to induce the formation of giant lipid droplets. Upregulation of *Plin2*, coding for the lipid coat protein Perilipin 2 (Plin2), has been reported to be associated with an upregulation of SNARE protein SNAP23, which promotes lipid droplet fusion^22^. This agrees well with our observation (Fig. 4) of increased expression of *Plin2* at fatty acid overload (both with and without co-treatment with Entinostat) where we saw a substantial increase in the number of large droplets (*r* > 0.8 μm), up to 8% and 13% for oleate alone and Entinostat co-treatments respectively, compared to just 2.7% for both the Entinostat only and control populations. While Plin2 has been reported to be localised later in the droplet formation process, on medium-sized droplets, Plin4 (also called S3-12) facilitates the very early stage formation of small lipid droplets^16^,^17^,^18^. The manifest upregulation of *Plin4*, for cells treated with Entinostat, both without, and even more strongly, with excess FFAs, correlates with the increased median number of droplets per cell we observe (121 and 199 for Entinostat and combined treatments respectively, compared to 96 at oleate overload): Plin4 appears then to be important for droplet formation in our sample under inhibition of HDACs. This observation is also in line with the qualitative observation of Sun *et al*., who noted the accumulation of small droplets with HDAC deletion^5^. The magnitude of the overexpression of *Plin4* (>eight-fold compared to the control), with Entinostat or combined treatment is so strong that perhaps we could expect to see even higher numbers of droplets seeded, and a possible reason we do not could be insufficient amounts of cholesterol esters, a minor component of hepatic lipids, but which have recently been noted to be preferentially associated with by Plin4^17^,^19^,^23^. Several studies have focussed on the role of Plin2 in liver steatosis^5^,^24^,^25^,^26^, whereas our results bring to light the potential importance of Plin4, as well as Plin2, and thus future studies of hepatic lipid storage mechanisms, and hence insulin sensitivity, could be more clearly understood taking into account Plin4 availability.

## Discussion

Over-nutrition results in an overload of different free metabolic intermediates known to be toxic and eventually leads to disruption of systemic insulin signalling^27^. However, if the intermediates are converted to triglycerides and sequestered as in perilipin-coated droplets in the liver, it has been shown that insulin resistance in mice can be reduced albeit with the development of hepatosteatosis^6^. *Hdac3* knock-out in the liver can promote the storage of lipids in droplets by rerouting metabolic precursors toward lipid synthesis and storage, resulting in higher insulin sensitivity^5^. This renders Class I HDAC inhibitors, such as Entinostat, interesting candidates for treatment of metabolic diseases and it highlights the need for better understanding of mechanisms behind the formation of hepatic lipid store droplets.

Beside modulation of the cell metabolism via gene expression, lysine acetylation has also been shown to be vital downstream. In response to different carbon sources, it controls the activation and inhibition of almost all cytoplasmic and mitochondrial enzymes involved in gluconeogenesis, glycolysis, the glycogen-, fatty acid- and nitrogen metabolism, assuring a more prompt feed-back loop, enabling the cell or organism to sense current energy status and immediately adapt to varying nutritional conditions^28^,^29^. Upregulation of the genes associated with lipid synthesis and sequestration (Fig. 4) allows us to conclude that the steatotic effect of Entinostat is caused at least in part, and perhaps primarily, by histone de-acetylation at the gene expression level^5^. However, we cannot rule out the possibility that lipid accumulation with Entinostat treatment occurs also due to de-acetylation of the enzymes in the glycolysis, gluconeogenesis, the tricarboxylic acid (TCA) cycle, fatty acid metabolism, and/or glycogen metabolism as during lipid load described by Zhao *et al*.^29^. A complete picture of the processes that lead to cellular lipid accumulation by Entinostat treatment could be derived from a high-throughput microarray for acetylation and deacetylation profiling and its correlation with a genome wide analysis of promoter bound lysine-acetylated-histones by ChipSeq. Whereas the primary target for Entinostat is gene regulation, the primary targets for oleate overload are enzymes regulating metabolism, as shown by the minimal response at gene expression level at oleate overload. Cell size differences (Fig. 2a) may be linked to the effect of lysine acetylation on cytoskeleton reorganisation^28^. Furthermore, as an HDAC1 (as well as HDAC3) inhibitor, Entinostat can have an effect on cell proliferation and growth. An investigation into the effects of Entinostat on lipid metabolism and insulin sensitivity in a diabetic mouse model carried out by Galmozzi *et al*.^30^ found, in contrast to our results, and another study^31^, no changes in hepatic gene expression after treatment, and lipid accumulation in the diabetic mouse model was cleared. Our results describe the effect of Entinostat in a non-transformed human hepatocytic cell line where features and properties of adult human hepatocytes are conserved, and hence could represent a better indication of the effect of HDAC inhibition on the human liver than transgenic animal models.

The decreased cell-to-cell variability with Entinostat treatment, with or without co-treatment with oleic acid, is marked: the interquartile range of the cellular lipid volume is significantly reduced in the presence of Entinostat (Fig. 2a,c), and the scatter of median droplet radius and droplet counts per cell is more uniform (Fig. 2e). Entinostat appears to induce lipid accumulation across all cells, overriding intrinsic cellular metabolic differences. This implies that it might have the capacity to relocate lipids already accumulated in the liver, storing them more safely, which bodes well for the possibility of its use in lipid storage induction as a treatment strategy.

In conclusion, we have shown that a specific HDAC inhibitor drug already in clinical trials for cancer treatment may be of use as a therapy for other epidemiologically important diseases. We see a particular pattern of uniform cell-to-cell increase in lipid droplet sequestration in a human liver cell model after incubation with Entinostat. The drug promotes the storage of metabolites as lipid droplets, both when the energy source is purely carbohydrates as well as at fatty acid overload; this provides a good basis for further investigation of Entinostat as a potential candidate for the treatment of diabetes and obesity. Upregulation of perilipin genes indicates that the droplets might be protected from immediate degradation, but the lipid morphology induced by the drug – small droplets, near to the nucleus – allows accessibility to lipolytic enzymes, which means the lipid storage is potentially reversible in the long-term. Intrahepatic fat accumulation, if left unchecked, can lead to the more advanced non-alcoholic steatohepatitis (NASH), which may progress to cirrhosis and, in a small percentage of patients, to hepatocellular carcinoma (HCC). However, only in a small percentage of patients (0–2.8%) will NASH progress to end-stage liver disease such as cirrhosis and HCC over a 19.5-year period, and therefore, as lipid accumulation is a reversible process it need not necessarily have a significant immediate negative impact on patient health^32^. Hence the induction of reversible safe lipid droplet accumulation in the liver by short-term administration of Entinostat or similar Class I HDAC inhibitors, temporarily and rapidly removing toxic free fatty acids from the cells and reducing the load of carbohydrates in the bloodstream that would otherwise interfere with insulin signalling, may provide a window of therapeutic opportunity to allow commencement of longer-term treatment strategies for obesity and metabolic disease such as changes in diet and exercise.

## Materials and Methods

### Cell preparation

Human hepatoma HepaRG cells were seeded at a density of 2.6 × 10^4^ cells/cm^2^ in William’s E medium with GlutamaX (GiBco) supplemented with 10% fetal bovine serum, 100 U/mL penicillin, 100 μg/mL streptomycin, 5 μg/mL insulin and 50 μM hydrocortisone hemi-succinate. In one week cells reached confluence with a doubling time of around 24 h and were shifted to the same medium supplemented with 2% dimethyl sulfoxide (DMSO) for a further two weeks to obtain confluent differentiated cultures^33^. After differentiation, HepaRG cells were incubated for 4 days with a solution of either sodium oleate (250 μM), Entinostat (8 μM), both sodium oleate (250 μM) and Entinostat (8 μM) or control. Three separate cell culture dishes were prepared for each treatment condition. After the period of incubation, the cells were fixed with 4% formaldehyde ready for imaging. The optimal conditions (dosage and incubation time) for fatty acid overload induction of vesicular steatosis in dHepaRG, as demonstrated by FACS analysis with BODIPY staining for neutral lipids, was established in preliminary experiments using 100, 250 and 500 μM of sodium oleate for 1, 2, 4 and 6 days. Entinostat dosage of 8 μM was chosen according to the manufacturer’s instructions (Selleckchem).

### CARS microscopy

A multimodal nonlinear microscope was used to record 3D stacks of images in the fixed cells, using the strong CH vibration at 2840 cm^−1^ of the lipids as image contrast. Details of the microscope may be obtained elsewhere^34^. In brief, a picosecond laser source (Levante Emerald OPO, APE Angewandte Physik & Elektronik GmbH, Germany, pumped by a Nd:Vanadate laser at 1064 nm, High Q Laser GmbH, Austria) generated two pulses at 76 MHz repetition rate, with powers of 50 mW and 120 mW for the pump (817 nm) and Stokes (1064 nm) beams, that were spatially and temporally overlapped and then coupled to a modified inverted laser scanning confocal microscope. A 40×/NA = 1.3 oil immersion objective was used to focus the light for imaging over a square field of 200 μm with lateral pixel size 0.098 μm (2048 × 2048 pixels) and create *z*-stacks with an axial spacing of 0.3 μm. The pixel dwell time was 9.6 μs. The CARS signal in the forward direction was collected with an aspheric lens (Thorlabs AL2520-B, NA = 0.54, WD = 15.7 mm) and detected using a photomultiplier tube connected to a time-correlated single photon counting unit (HPM-100-40 connected to an SPC-150 module card, Becker & Hickl GmbH, Germany) after filtering appropriately: first a long pass dichroic mirror (FF775-Di01, Semrock Inc., USA) was used to separate the excitation beams from the CARS signal (emitted at 663 nm) followed by two short pass 790 nm filters (Semrock FF01-790/SP) blocking any remaining reflected excitation light and finally a band pass filter centred at 661 nm with 20 nm of bandwidth (Semrock FF01-661/20) to further clean and eliminate any fluorescence emitted by the sample.

### Image processing and analysis

All image processing and analysis was carried out using the Fiji implementation of ImageJ, supplemented also by MATLAB scripts. In total, three-dimensional *z*-stacks covering the entire volume of a total of 267 control cells, 165 cells treated with Entinostat, 230 cells treated with oleate, and 167 cells treated with the combination of oleate and Entinostat were imaged. The experiments were conducted with three replicates for each of these categories (three separate dishes per category). Analysis was performed on >50 cells from image stacks of each of the three replicates of each treatment condition: *n* = 85, 102, 80 for control cells; *n* = 72, 81 and 77 for oleate treated cell; *n* = 52, 59 and 56 for combined oleate and Entinostat treated cells; and *n* = 53, 61 and 51 for Entinostat treated cells. These cell counts represent all the cells imaged in each of three images (for control and oleate treated cells) or five images (for combined oleate and Entinostat and Entinostat only treatments).

The images were corrected for saturation of a few pixels using the known dead time of the detection unit (MATLAB). Before carrying out analysis of the lipid structures in the cells, the *z*-stacks of images were smoothed using the PureDenoise algorithm plugin for ImageJ^35^, which allowed effective removal of noise, and then corrected for differences in the laser power.

In order to obtain cell-to-cell statistics on the lipid content, image stacks were laterally divided into stacks for single cells. This also facilitated the optimal selection of threshold for the volumetric and area analysis, which was slightly dependent on the position within the field of view (Fiji ImageJ). For the single cell analysis in 3D, the 3D object counting routine in ImageJ was run on every cell separately, to retrieve volumes for all droplets which were summed to give the cellular lipid volume. For some data, such as the lipid droplet counts, a 2D analysis, which included using a watershed algorithm to effectively discriminate adjacent particles was used (see Supplementary Information for more details on this aspect). For the 2D analysis, selection of a single *xy* image from the brightest section of each cell was followed by segmentation of each cell into droplets and background by thresholding, setting the threshold by inspection of each cell separately (Fiji ImageJ). The Watershed function in Fiji ImageJ was also used, allowing the counting of separate adjacent lipid droplets by subsequent use of the ImageJ Measure function from which the droplet areas and centroid coordinates were also obtained. These were used to compute the droplet counts per cell, and the droplet radii, as well as the distance of droplet centroids from the nuclei; some justification of the use of single plane images for the computation of these quantities is given in the Supplementary Information and in Supplementary Figs 5–7. Cell area masks and cell nuclei masks were also determined from inspection of the 2D plane images for each cell. These were then used to compute the cell area and the droplet to nucleus edge distance. Radii for cells, nuclei and droplets were all computed as averages from the measured areas of these particles. For both the single plane and 3D counting, no lower limit on the number of pixels per droplet was set.

Box-and-whisker plots, scatter plots and histograms (Figs 2 and 3) were created using Origin. For each population in all the box-and-whisker plots presented here, box upper and lower limits are the 25th and 75th percentiles, the median is the horizontal line through the box, the mean is thicker horizontal line, and the upper and lower limits of the vertical whiskers are ±1 standard deviation of the mean.

### PCR analysis

Total cellular RNAs were extracted from cells treated as described above using TRIzol reagent (Gibco BRL), and 1 μg was reverse transcribed with the ThermoScript reverse transcription (RT)-PCR system (Invitrogen). cDNAs were quantified by real-time PCR analysis (Light Cycler; Roche Diagnostics) using gene specific primers shown in the Table in Supplementary Fig. 8. The 18S ribosomal RNA was used to normalize the RNA samples. Relative expression was calculated using the comparative Ct method (2^−ΔCp^, ΔCp = Cp (target gene) - Cp (18S)). Data are expressed as fold induction (treated vs untreated) of 2^−ΔCp^ mean (Fig. 4).

## Additional Information

**How to cite this article**: Nunn, A. D. G. *et al*. The histone deacetylase inhibiting drug Entinostat induces lipid accumulation in differentiated HepaRG cells. *Sci. Rep.* **6**, 28025; doi: 10.1038/srep28025 (2016).

## Supplementary Material

Supplementary Information
