# Erratum: The histone deacetylase inhibiting drug Entinostat induces lipid accumulation in differentiated HepaRG cells

**DOI:** 10.1038/srep37204

**Published:** 2016-11-28

**Authors:** Abigail D. G. Nunn, Tullio Scopigno, Natalia Pediconi, Massimo Levrero, Henning Hagman, Juris Kiskis, Annika Enejder

Scientific Reports 6: Article number: 28025; 10.1038/srep28025 published online: 06 20 2016; updated: 11 28 2016

In the original Supplementary Information file published with this Article, the y-axis label for Supplementary Figure 2 was omitted. This has now been updated to read ‘Mean Fluorescence Intensity’.

This error has been corrected in the Supplementary Information that now accompanies the Article.

