# Supplementary material for: The histone deacetylase inhibiting drug Entinostat induces lipid accumulation in differentiated HepaRG cells
Source: Sci Rep. 2016 Jun 20;6:28025. doi: 10.1038/srep28025 (PMC4913258; doi:10.1038/srep28025)
Supplement: Supplementary Information [file srep28025-s1.pdf]

Supplementary Information for *The histone deacetylase inhibiting drug Entinostat induces lipid accumulation in differentiated HepaRG cells, A. D. G. Nunn, T. Scopigno, N. Pediconi, M. Levrero, H. Hagman, J. Kiskis, and A. Enejder*

Cytotoxicity testing. Cytotoxicity of the different treatments was assessed by two complementary methods, the colorimetric 3-(4, 5-Dimethyl-2-thiazolyl)-2, 5-diphenyl-2H-tetrazolium bromide (MTT) assay[1], which measures the activity of mitochondrial dehydrogenase enzymes in living cells, and the sulforhodamine B (SRB) assay[2], that relies on SRB stoichiometric binding to cell proteins in tri-chloroacetic acid fixed cells to measure the cell mass. For the MTT assay, water soluble yellow MTT is converted in metabolically active cells to water insoluble purple formazan that can be extracted by dimethyl sulfoxide (DMSO). MTT (Sigma) solution at 1 mg/ml in complete William's E medium was added to HepaRG cells grown in 24 wells cell plates and treated or left untreated as described in Materials and Methods, in the main text. After 3 hours at 37°C, 5% CO_2, 95% air and complete humidity, the MTT solution was removed, replaced with 500 µl of DMSO at room temperature. The plates were further incubated for 5 min and the optical density (OD) of the cells was then determined at a wavelength of 570 nm. For the SRB assay, the stoichiometric binding of the two sulfonic groups present in the bright pink SRB amino-xanthene dye to basic amino-acids under mild acidic conditions is reversed under basic conditions and the amount of dye extracted is directly proportional to cell mass. After addition of 0.1 mL/cm^2 of SRB solution at 1 mg/ml, cells were incubated for 5 min at room temperature, washed three times with 0.3 mL/cm^2 of 1% acetic acid solution and completely dried at 37 °C. SRB was extracted by gentle shaking for 5 min with 0.3 mL/cm^2 of 10 mM Tris pH 10.5 and the optical density was measured at 490 nm. Results of these assessments are shown in Supplementary Fig. 1.

FACS analysis. We show by fluorescence activated cell sorting (FACS) analysis using BODIPY staining, a lipophilic bright green fluorescent dye for detection of intracellular non-polar lipids, that lipids accumulated in the cells after Entinostat treatment at the same level as oleic acid treatment

alone (Supplementary Fig. 2). Together, these results support our CARS image analysis in suggesting that inhibition of HDAC causes de-repression of metabolic genes that activate lipid synthesis and intracellular sequestration, which underlies hepatic lipid accumulation.

Cell and nuclear sizes. A direct comparison of the droplet to nuclear distances between the different treatment types is only possible if there is no significant variation in nuclear size, and of course the cell dimension, which is clearly different for the treatments, must also be considered when making the comparison. In the table in Supplementary Fig. 3 we show averages and ranges for the droplet to nucleus distances alongside those for the cell area (full distributions shown in Supplementary Fig. 4) and nuclear radius (the nuclear radius is the averaged value computed from the nuclear area). In fact, we see that the nuclear radii do not vary significantly between treatments, despite the large change in cellular area.

Three-dimensional versus two-dimensional image analysis. Three dimensional analysis clearly provides a more rigorous way to quantify the spatial amounts of subcellular organelles in general than simply relying on single plane images. However, it must be noted that our 3D image analysis procedure (illustrated in Supplementary Fig. 5) may underestimate the total number of individual lipid droplets, as the thresholding routine used to compute the droplet count in 3D was set to a level optimised to select as much as possible of the cellular lipid volume; this was carried out with the ImageJ 3D Object Counter, which does not include any watershedding, and as a result, lipid droplets organized in dense clusters are counted as a single lipid droplet and therefore the droplet counts from the 3D image stacks, as shown in Supplementary Fig. 6, probably represent underestimations in cases where the clustering of droplets in the cells is strong. For this reason, droplet statistics from single plane analysis are presented in Figs. 2b, 2e-i and 3 (Fig. 2d combines single plane statistics for the droplet counts and 3D analysis for the volumes) rather than from volumetric analysis.

In two dimensions, cell boundaries are clear, and hence provide the capacity for both the cell area quantification as presented in Fig. 2a and Supplementary Fig. 4 and the nuclear edge definition used to generate the data in Fig. 3. Some justification of our use of 2D single plane analysis methods is

given in Supplementary Fig. 7, in which we plot the interquartile range (IQR) of the droplet axial coordinate values from the 3D object counting results for each cell. The small range of values in this plot (the 75th percentile for each treatment case is less than 2 μm) compared to the size of the droplet radii (distributions peaking around 0.4 μm, see Figs. 2f-i) is quantitative support of our observation of the flatness of the cells.

Statistics and variation. The minimal variation between dishes in this experiment is illustrated by the fact that for all treatments, the median of total lipid volume in each of the three dishes varied by no more than 40% from the global median. In Supplementary Figs. 4, 6 and 7 all the box-and-whisker plots, as those in the main text, show with boxes the 25th, 50th and 75th percentiles for the full cell dataset for each treatment, and the mean and ±1standard deviation are indicated by the thick horizontal line and the whiskers respectively. The colour code in Supplementary Figs. 1-4, 6 and 7 is also as in the main text, with pink for the control cells, green for the Entinostat, blue for the oleate and cyan for the combined treatments.

Supplementary References

1. Plumb, J. A., Milroy, R. & Kaye, S. B. Effects of the pH Dependence of 3- (4 , 5-Dimethylthiazol-2-yl) -2 , 5-diphenyl- tetrazolium Bromide-Formazan Absorption on Chemosensitivity Determined by a Novel Tetrazolium-based Assay1. *Cancer Res.* 4435–4440 (1989).

2. Vichai, V. & Kirtikara, K. Sulforhodamine B colorimetric assay for cytotoxicity screening. *Nat. Protoc.* **1,** 1112–1116 (2006).

Supplementary Figures

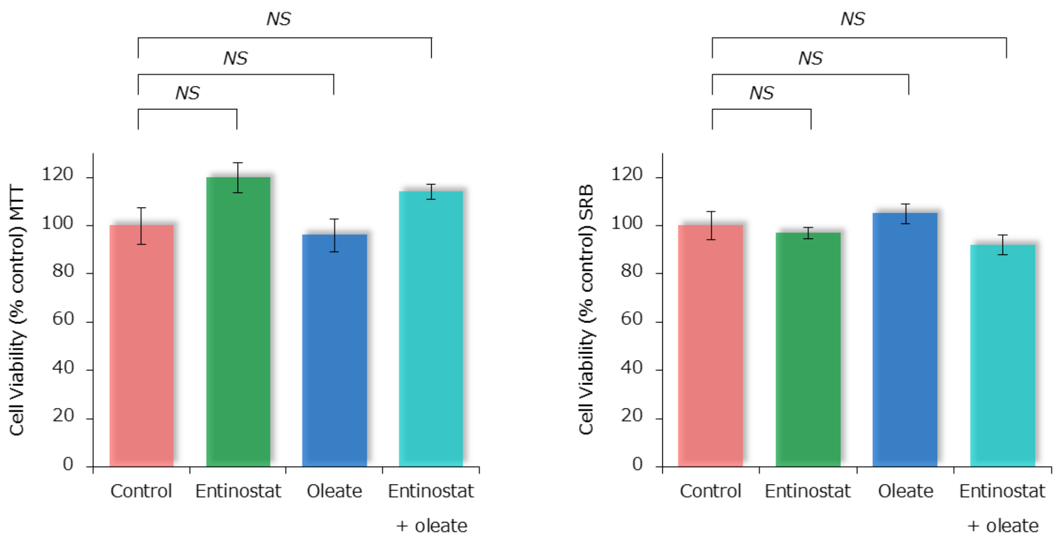

Supplementary Figure 1. Cytotoxicity of Entinostat, oleate and Entinostat + oleate in dHepaRG cells. Cytotoxity for both the MTT assay (left) or the SRB assay (right) is expressed as % relative to controls. Mean (bar height) and standard deviation (half the inlaid line on each bar) for each treatment are shown. Horizontal lines above the bars represent pairs with differences that are not significant (NS).

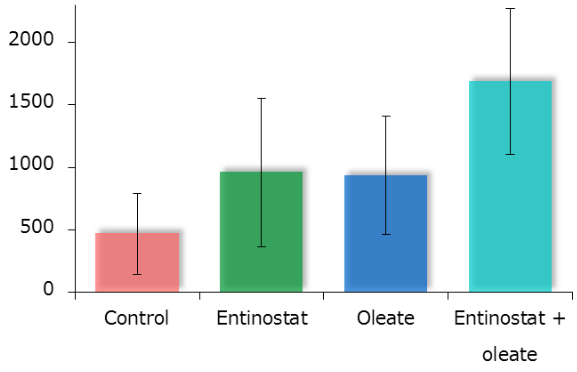

Supplementary Figure 2. FACS analysis using Bodipy. Two repeats were used to quantify mean (bar height) and standard deviation (half the inlaid line on each bar) of non-polar lipid content in cells for each treatment as shown.

	Droplet-nucleus distance for all droplets in cells in sample (μm)		Radius of nucleus (μm)		Area of cell (μm²)	
	Median	*IQR/2*	*Median*	*IQR/2*	*Median*	*IQR/2*
Control	5.13	3.82	6.51	0.54	809	280
Entinostat	6.35	4.27	6.74	0.44	1435	405
Oleate	8.00	4.95	6.44	0.46	902	259
Entinostat + oleate	6.83	4.26	6.48	0.57	1285	378

Supplementary Figure 3. Table showing the comparison between cell and nuclear size with the distances between the centroids of the droplets and the edge of the nucleus. For the droplets, the median and IQR are computed over all droplets in the full cell population sample.

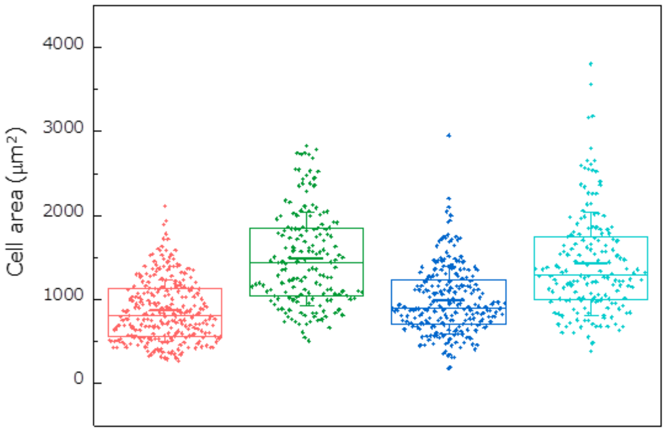

Supplementary Figure 4. Distribution of cell areas for the different treatments.

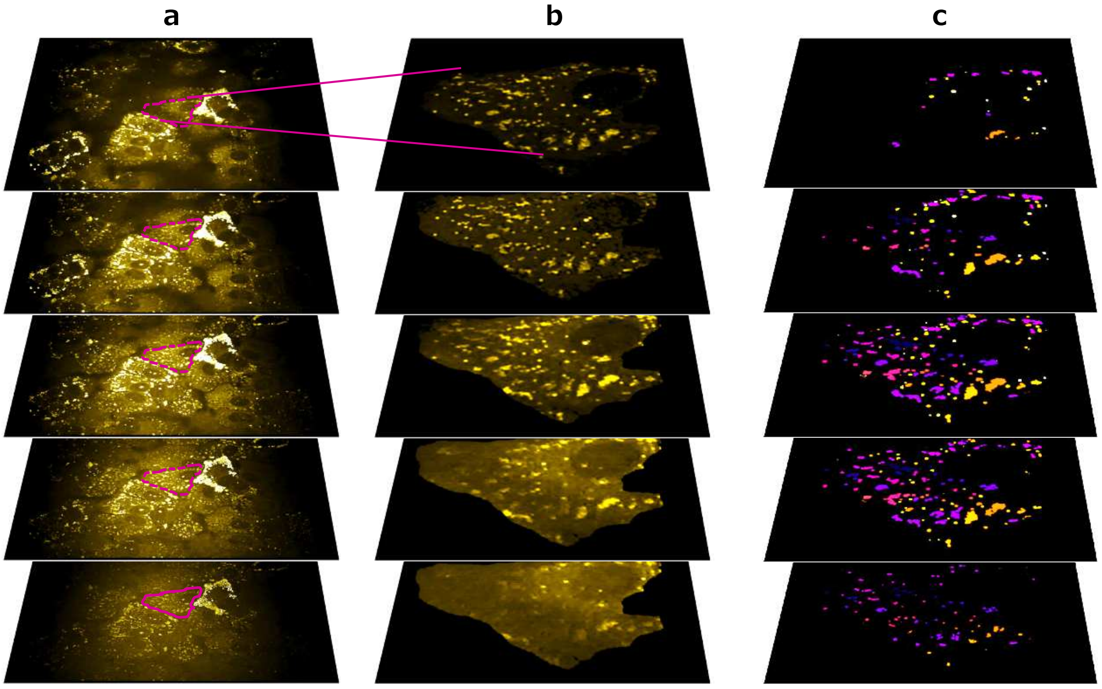

Supplementary Figure 5. Illustration of the analysis method used on individual *z*-stacks of all cells. In this example, the cells were overloaded with oleate. First the *z*-stack of images (**a**) is divided into cells, taking a single slice near the base of the stack where the cell boundaries are clearest and creating a mask of the shape of the cell boundary. The single cell *z*-stack (**b**) undergoes thresholding and 3D object counting and the result (**c**), in this example 145 separate droplets, is used again as a mask to count volumes of droplets (summing the number of pixels in each 3D droplet). The colours shown in (**c**) are used to visually differentiate droplets as produced by the counting routine: colours are assigned in counting order, starting from the first droplet that appears in the uppermost part of the image that forms the lowest slice in the stack using the “fire” LUT in ImageJ.

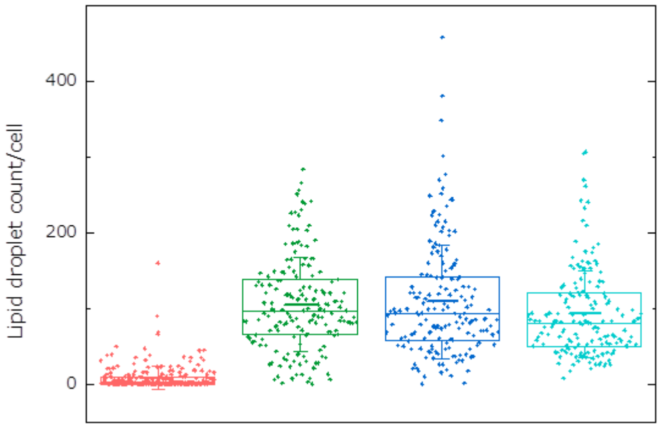

Supplementary Figure 6. Droplet counts per cell computed from the 3D object counting results.

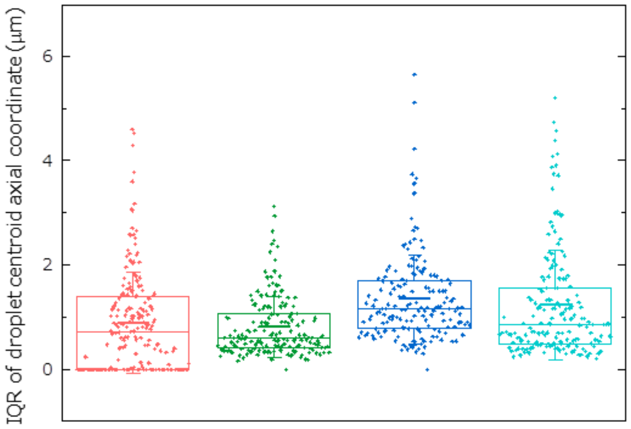

Supplementary Figure 7. Plot of the distribution of ranges of axial (*z*) coordinate in each cell. For all treatments and the control, the 75th percentile of the IQR of the droplet *z* coordinate is less than 2 µm.

Plin2 for	TTGCAGTTGCCAATACCTATGC
Plin2 rev	CCAGTCACAGTAGTCGTCACA
Cidec for	CAGCAGCTCCTCGATGCTAC
Cidec rev	TCAGACAGGTCGGGATAAGGG
Acacb for	CAAGCCGATCACCAAGAGTAAA
Acacb rev	CCCTGAGTTATCAGAGGCTGG
Gpam for	TCTTTGGGTTTGCGGAATGTT
Gpam rev	ATGCACATCTCGCTCTTGAATAA
Scd for	TCTAGCTCCTATACCACCACCA
Scd rev	TCGTCTCCAACTTATCTCCTCC
Fasn for	AACTCCTGCAAGTTCTCC
Fasn rev	GCTCCAGCCTCGCTCTC
Plin4 for	GAAGGAGCTGCAACCTTCGGAAAAG
Plin4 rev	TGGACCACTCCCTTAGCCAC

Supplementary Figure 8. qPCR oligos.
